# Management Outcomes of Large Renal Angiomyolipoma Presenting with Wunderlich Syndrome—Experience from a Tertiary Center

**DOI:** 10.15586/jkcvhl.v10i2.265

**Published:** 2023-06-06

**Authors:** Atanu Kumar Pal, Sidhartha Kalra, Sreerag Kodakkattil Sreenivasan, Lalgudi Narayanan Dorairajan, Ramanitharan Manikandan, Shailendra Kumar Sah

**Affiliations:** Department of Urology & Renal Transplantation, Jawaharlal Institute of Post Graduate Medical Education and Research (JIPMER), Gorimedu, Puducherry, India

**Keywords:** angioembolization, angiomyolipoma, nephrectomy, perirenal hemorrhage, tuberous sclerosis, Wunderlich syndrome

## Abstract

Renal angiomyolipoma is an uncommon, benign-mixed mesenchymal tumor consisting of thick-walled blood vessels, smooth muscles, and mature adipose tissues. Twenty percent of these tumors are associated with tuberous sclerosis. Wunderlich syndrome (WS), an acute nontraumatic spontaneous perirenal hemorrhage, can be a presentation of large angiomyolipoma. This study evaluated the presentation, management, and complications of renal angiomyolipoma with WS in eight patients who presented to the emergency department between January 2019 and December 2021. The presenting symptoms included flank pain, palpable mass, hematuria, and bleeding in the perinephric space on computerized tomography. Demographic data, symptoms at presentation, comorbidities, hemodynamic parameters, the association with tuberous sclerosis, transfusion requirements, need for angioembolization, surgical management, Clavien–Dindo complication, duration of hospital stay, and 30-day readmission rates were evaluated. The mean age of presentation was 38 years. Of the eight patients, five (62.5%) were females and 3(37.5%) were males. Two (25%) patients had tuberous sclerosis with angiomyolipoma, and three (37.5%) patients presented with hypotension. The mean packed cell transfusion was three units, and the mean tumor size was 7.85 cm (3.5–25 cm). Three of them (37.5%) required emergency angioembolization to prevent exsanguination. Embolization was unsuccessful in one patient (33%) who underwent emergency open partial nephrectomy, and one (33%) patient developed post-embolization syndrome. A total of six patients underwent elective surgery—four underwent partial nephrectomy (laparoscopic - 1, robotic - 1, open - 2) and two underwent open nephrectomy. Three patients encountered Clavien–Dindo complications (Grade 1, n = 2 and IIIA, n = 2). WS is a rare, life-threatening complication in patients with large angiomyolipoma. Judicious optimization, angioembolization, and prompt surgical intervention will help deliver better outcomes.

## Introduction

Angiomyolipomas (AMLs) are uncommon, though the most frequent being benign renal tumor, with a prevalence rate varying between 0.2 and 0.6% (1). It is a mesenchymal neoplasm of the kidneys, composed of variable proportions of mature adipose tissue, smooth muscles, and abnormal blood vessels. Eighty percent of the renal AMLs occur as sporadic cases. The remaining 20% develop in association with tuberous sclerosis complex (TSC) or pulmonary lymphangioleiomyomatosis (LAM). It is most commonly detected as an incidental finding (80%) with the recent advent of more cross-sectional imaging of the patients. In 15% of cases, renal AMLs present with spontaneous retroperitoneal hemorrhage or Wunderlich syndrome (WS). At least one-third of these patients present with shock (2). TSC-associated renal AMLs are more likely to be large, multifocal, and complicated by flank pain, abdominal pain, spontaneous retroperitoneal hemorrhage, or hematuria (3). Thus, the clinical concern of a life-threatening hemorrhage is present in patients with AML.

Wunderlich syndrome, first described by Carl Reinhold August Wunderlich in 1856, is an uncommon and potentially fatal condition resulting from an acute onset of spontaneous, nontraumatic perirenal hemorrhage spreading into the subcapsular and perinephric spaces. WS is characterized by Lenk’s triad consisting of acute flank or abdominal pain, palpable flank masses, and ultimately fulminant hypovolemic shock, but the clinical symptoms vary and are nonspecific (4). AML and renal cell carcinoma represent the most common benign and malignant neoplasms that cause this syndrome, respectively. Nonmalignant and nonrenal causes such as adrenal, aneurysm, and vasculitis are also there. Ultrasound (US), multidetector computed tomography, and magnetic resonance imaging (MRI) play a significant role in the detection of perinephric hemorrhage and characterization of the myriad causes of WS and thus provide a roadmap for the management of WS (5). Besides conservative management and embolization, surgical management has a definitive role in managing WS due to renal AMLs.

There is a paucity of literature regarding the clinical presentation and management of WS due to renal AMLs due to the rarity of this condition. In this study, we have assessed the demographics, presentation, and different management strategies of large renal AMLs presented in the emergency with WS.

## Materials and Methods

Eight patients were included in this study who presented to the emergency department between the period of January 2019 and December 2021. Ethical approval for this study was obtained from the Institutional Review Committee. Patients who presented to the emergency department with symptoms of AML and imaging evidence of AML with perirenal hemorrhage (WS) and required intervention were included in the study. A total of 15 patients presented with suspicion of Wunderlich syndrome during the above period. Seven patients were not counted in the study as the causes of bleeding were not renal angiomyolipoma. Renal AML patients with no symptoms, renal AML with no evidence of perirenal hemorrhage (WS), patients with renal mass and perirenal hemorrhage with causes other than renal angiomyolipoma, and those who did not give their consent were excluded from the study.

All the patients came up with sudden onset flank pain. Initial history and clinical examination were done after stabilizing the patients. Bedside US was done to rule out other causes of acute onset flank pain. All the patients were subjected to routine blood work. All except one patient underwent a contrast-enhanced CT scan of the abdomen. After taking the non-contrast film, sequential dynamic images were taken in arterial, venous, excretory, and delayed phases at 25–80 s, 80–120 s, 2–5 min, and 7–10 min, respectively. Iohexol was used as low osmolality contrast media. The perinephric hematoma was defined with > 60 Hounsfield units (HU) in the perinephric space which signified bleeding. The fat component of the renal AMLs was characterized by < 10 HU. Only non-contrast CT was done in the patient with raised serum creatinine level. Significant past medical history, hemodynamic stability, and preintervention blood transfusion requirement were noted.

After the diagnosis, the patients underwent management through angioembolization and surgery. Surgical management was done by open, laparoscopic, and robot-assisted partial or radical nephrectomy. In the postoperative period, the complication in Clavien–Dindo classification, duration of hospital stays, and 30-day readmission rates were assessed. The evaluation for TSC was done in those patients in which it was not done before. In all the operated cases, specimens were sent for histopathological examination for confirmation of the diagnosis of angiomyolipoma.

Informed consent was obtained from all the patients included in this study. No information has been included in this article that would reveal the identity of any patient.

## Results

A total of eight patients were included in this retrospective study. Five were males (62.5%), and three were females (37.5%). The mean age at presentation was 38.5 ± 10.4 years. All the patients presented with flank pain (four cases had left, three cases had right, and one case had bilateral flank pain); however, Lenk’s triad was present in only three cases (37.5%). Only two patients had symptoms of gross hematuria. Three patients had hypertension, two patients had diabetes, and one had a history of chronic kidney disease (CKD) not requiring dialysis. Two patients were diagnosed case of tuberous sclerosis. One patient had a previous history of Wunderlich syndrome 2 years back, which was managed conservatively (Table 1).

**Figure 1:**
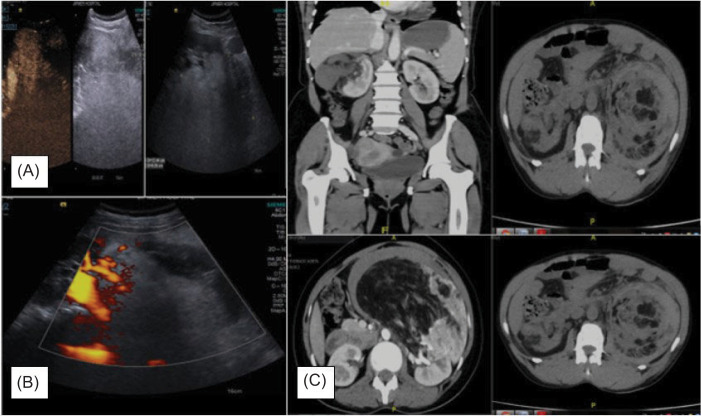
(A) Ultrasonographic images of renal angiomyolipoma; (B) Doppler images showing perirenal haemorrhage, that is, Wunderlich syndrome; (C) Contrast-enhanced computed tomography showing renal angiomyolipoma with Wunderlich Syndrome.

On CT scan, the size of the tumor varied at a wide range, with the smallest AML being 5.5 × 4.5 × 5 cm and the largest being 25 × 15 × 12 cm. CT scan was effective in both perinephric hematoma and AMLs in all the cases (Figure 1). Two of the patients had bilateral AML (Table 2).

**Table 1:** Demographics, symptoms and blood investigations.

Parameters (n = 8 patients)		Observed value
Age		38.5 ± 10.4 years
Sex	Male	5 (62.5%)
	Female	3 (37.5%)
Comorbidities	Hypertension	3 (37.5%)
	Diabetes	2 (25%)
	CKD	1 (12.5%)
Symptoms	Flank pain	8 (100%)
	Gross hematuria	2 (25%)
	Fever	1 (12.5%)
	Lenk’s triad	3 (37.5%)
Association with tuberous sclerosis		2
Previous history of Wunderlich syndrome		1
Blood report	Mean hemoglobin	6.3 gm/dL (Range 4.8–8.2 g/dL)
	WBC	11.7/mm^3^
	Platelet	178,000/mm^3^

**Table 2:** Computed tomography (CT) imaging characteristics of angiomyolipoma (8 cases).

Tumour characteristics (n = 8 patients)		Observed value
Laterality	Right	3 (32.5%)
	Left	5 (67.5%)
	Bilateral	2 (25%)
Location	Upper	5 (67.5%)
	Middle	2 (25%)
	Lower	1 (12.5%)
Mean lesion size (cm)	7.85 × 8.5 × 10.4	
Maximum/Minimum lesion size (cm)	25 × 15 × 12/6.5 × 5.7 × 4.8	

At presentation, three patients had shock with blood pressure below 90/60 mm of Hg. The mean pulse rate was 90/min. The mean hemoglobin level was 6.3 g/dL (range 4.8–8.2 g/dL). The serum creatinine value of all the patients was within the normal limit (reference level 0.5–1.5 mg/dL) except in one patient whose serum creatinine level was 4 mg/dL. Preintervention blood transfusion was done in seven patients, with a mean transfusion of 2.56 units (range 1–5 units) (Table 3).

**Table 3:** Management strategies of the patients.

Management plan (n = 8 patients)	Findings
Patients presented with shock (BP <90/60 mm of Hg)	3 (37.5%)
Preoperative transfusion requirement (units)	2.56 (Range 1–5)
Patients underwent selective artery angioembolization (SAE)	3 (37.5%)
Patients underwent surgical management	6 (75%)
Patient underwent surgical management after failing angioembolization	1
Mean duration from admission to angioembolization suite (hour)	28
Mean duration from admission to surgery (hour)	40

**Figure 2:**
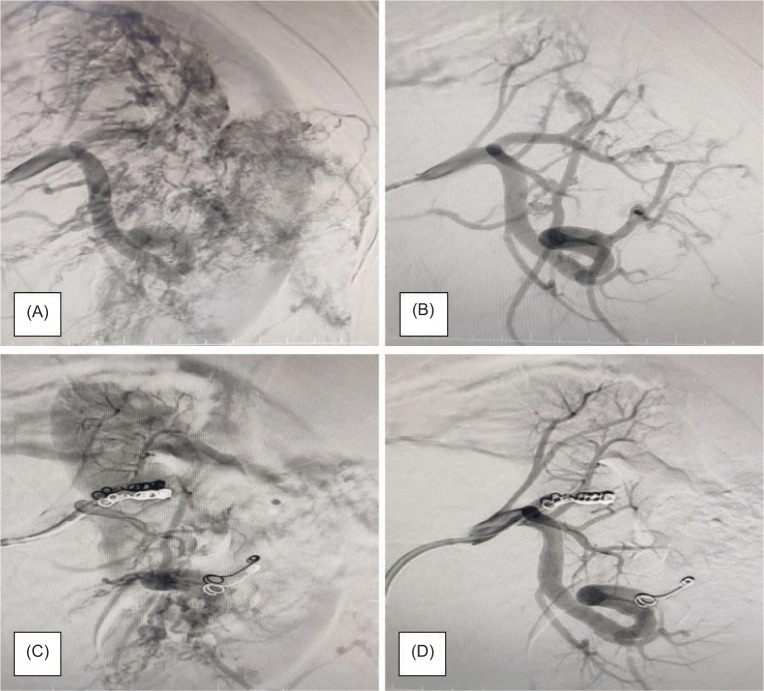
(A & B) Angiography showing the renal vasculature in a patient with renal angiomyolipoma; (C & D) Selective angioembolization showing the coils in place.

All patients underwent intervention after initial stabilization according to the standard ICU protocol. Three patients underwent emergency angioembolization, and three coils were applied in each case (Figure 2). Two of them developed post-embolization syndrome with post-procedure fever and nausea managed conservatively. One of them continued to have flank pain with a serial drop in hemoglobin level even after embolization that led to an open partial nephrectomy ultimately (Table 4).

**Table 4:** Patients who underwent selective angioembolization (SAE) – 3 patients.

Angioembolization findings (n = 3)	Number	Comments
Number of coils deployed	3 (each case)	
Post-embolization syndrome	2 (66.6%)	Managed conservatively
Failure	1 (33.3%)	Underwent surgery
30-day readmission	Nil	

Surgical intervention was done in six cases, including one failed angioembolization. An open approach was taken in four patients. Two patients underwent laparoscopy, and one of them had robot-assisted laparoscopy. Partial nephrectomy was done in four cases. In the other two cases, kidney could not be salvaged, and complete nephrectomy was done. Adhesions in the perinephric region along with altered anatomy were faced intraoperatively, and average time of open nephrectomy was 185 min. A perioperative blood transfusion was done in two cases. One of the partial nephrectomy cases developed urinoma for which pigtail drainage and double J stenting were done. One of the patients who underwent an open nephrectomy had a wound infection, and secondary suturing was done, in Clavien–Dindo classification, in four patients who encountered complications (Grade 2: n = 2, Grade IIIA: n = 2). Thirty days of readmission was present in only one patient (Table 5).

**Table 5:** Patients who underwent surgical management (6).

Surgical management: Findings (n = 6 patients)	Number	Comments
Open nephrectomy	4 (66.6%)	Post op transfusion – 2 cases (mean 1.5 units) Post-op Ileus – 2 (50%)
Laparoscopic nephrectomy	2 (33.3%)	Post op blood transfusion - Nil Post-op Ileus - Nil
Robot-assisted laparoscopic nephrectomy	1 (16.6%)	
Partial nephrectomy	4 (66.6%)	Mean Ischemia time 32 min
Complete nephrectomy	2 (33.3%)	
Clavien–Dindo complication	4 (66.6%)	Grade 2 – 2 Grade 3A - 2
30-days readmission	1 (16.6%)	
Mean post-op stay	Open – 5 days	Lap – 2.5 days

When we compared the angioembolization group (n = 3) with the surgically treated group (n = 5) we found that angioembolization group had less BP (88/64 vs 114/78 mm of Hg), more pulse (102 ± 7.2 vs 87.8 ± 6.5/min), less hemoglobin at presentation (5.9 ± 1.3 vs 7.1 ± 1.6 gm/dL), more transfusion requirement (3 vs 1.6 unit) with large sized tumors (13 ± 3.5 vs 7.5 ± 4.2 cm in largest dimension) (Table 6).

**Table 6:** Comparison between the patient group who underwent selective angioembolization (n = 3) and the patient group who underwent surgical management (n = 5).

Parameters	Patients underwent selective angioembolization (n = 3)	Patients underwent surgical management (n = 5)
Age (years)	42.6 ± 14.3	36 ± 9.6
Male: Female	1:2	3:2
Presentation: Lenk’s triad (no. of patients)	2/3	1/5
Location	Upper: 2; Middle 1	Upper: 3; Middle 1; Lower: 1
Mean Pulse	102 ± 7.2	87.8 ± 6.5
Mean BP	88/64	114/78
Hemoglobin at presentation	5.9 ± 1.3	7.1 ± 1.6
Blood transfusion (Mean: unit)	3	1.6
Mean size	13 ± 3.5	7.5 ± 4.2
ICU admission (no. of patients)	3/3	3/5

All post-operative specimens showed angiomyolipoma in histopathological examination. In a mean follow-up period of 12 months, no patient died. Only one patient with tuberous sclerosis and multiple AMLs developed recurrence.

## Discussion

Angiomyolipoma is a benign renal tumor manifested in middle-aged women. They tend to be single and small and rarely progress to cause significant morbidity when sporadic (6). In 20% of the cases, AMLs are associated with TSC or, less commonly, as part of LAM. They are multiple, large, bilateral, and more aggressive, affecting the younger population of both genders equally (3). AML occurs in association with 75% cases of TSC. The size criteria 4 cm for the intervention is now being questioned. Besides symptoms, size, presence in women of childbearing age and poor access to follow-up intralesional aneurysmal diameter are also being considered now for intervention (7,8).

Bonet first described the spontaneous retroperitoneal hemorrhage due to the rupture of the renal parenchyma. In 1856, Wunderlich carried out the first clinical description of this condition. In 1910, Coenen coined the term “Wunderlich Syndrome” to retroperitoneal hemorrhage without trauma or prior urological or vascular manipulation (9). In a literature review in 1975, Mcdougal et al. identified tumors as the first cause of WS, with renal AMLs as the commonest among them (10). The most recent meta-analysis in 2002, conducted by Zhang et al., reviewed 165 patients with WS between 1985 and 1999. The renal neoplasm was the most common cause of WS, with AML being the most common neoplasm (11). Other causes of this rare syndrome include vascular abnormalities (polyarteritis nodosa [PAN], aneurysms, arteriovenous malformations [AVMs] and fistulas [AVFs], and venous thrombosis), hereditary and acquired renal cystic diseases, renal infections, calculus disease, pheochromocytoma, retroperitoneal tumors, and coagulation disorders (12). In this study, we have focused only on AML as the cause of WS and its presentation to the management.

In the meta-analysis, Zhang et al. showed that 83% of the patients presented with acute onset of flank pain, 19% had hematuria, and 11% had symptoms and signs of hypovolemic shock (11). In another series, classical Lenk’s triad was seen in only 20% of the cases (13). In our study, all the patients presented with flank pain, but only three of them (37.5%) presented with symptoms of Lenk’s triad (Table 1).

In all cases presenting to the emergency department, ultrasound, CT, and MRI help to identify the perinephric hematoma and the cause of bleeding (14). The CT features of AML include a well-defined heterogeneous lesion with varying degrees of macroscopic fat, hypervascular soft tissue components, and intralesional aneurysms (15). The chance of rupture and bleeding increases with the increased size (>4 cm) (7). Contrast-enhanced CT scan is a standard medical imaging modality with 100% sensitivity in identifying perirenal haemorrhage (16). Zagoria et al. commented that CT combined with MRI is accurate for diagnosing spontaneous perinephric hemorrhage. Still, the underlying pathological condition is often undetectable in the acute phase due to the perinephric blood (17). In our study, a CT scan was used as a diagnostic modality in all the cases (Table 2).

There have been no prospective, randomized trials comparing surveillance versus treatment for AML. Ouzaid et al. published the largest series of AMLs managed with active surveillance including 130 patients, in which almost 80% of them were asymptomatic and 29% had masses >4 cm; and with 34% of the patients with lesions >4 cm having undergone treatment (18). However, there is no consensus regarding large symptomatic renal AMLs presenting to the emergency.

Recently, a more significant trend has been observed in managing renal AMLs with angioembolization especially in cases of acute hemorrhage and refractory hemodynamic instability. Recurrence rates are highly variable, ranging from approximately 11–40% (19). Complications from selective artery angioembolization include post-embolization syndrome, vascular injury, renal infarction with abscess formation, and nontarget embolization (20). Post-embolization syndrome, characterized by fever, flank pain, and leucocytosis, is the most common, with reports of occurrences in up to 80% of cases, and is managed conservatively (19). In our study, three patients underwent emergency angioembolization. Two of them developed post-embolization syndrome with post-procedures that were managed conservatively. One of them continued to have flank pain with a serial drop in hemoglobin level, ultimately leading to surgery (Table 4).

Surgical management remains the well-characterized form of management when indicated. Over time, surgery for AML has progressed from open and complete nephrectomy to nephron-sparing surgery (NSS) with a minimally invasive approach. NSS is particularly useful when there is a solitary kidney and bilateral with multifocal AML in patients with TSC. Boorjian et al. included 58 patients in their series of NSS in AML patients. They demonstrated at a median follow-up of 8 years a 3.4% recurrence rate and 12% complication rate with no de novo chronic renal insufficiency (21). Minervini et al. in a series of 34 AMLs treated with open NSS found that increased tumor size correlated with increased intraoperative estimated blood loss, ischemia time, and duration of hospital stay. The most commonly encountered complications following NSS for AML are urinary leak or fistula, hemorrhage, and ileus (22). In our study, surgical intervention was done in a total of six cases. In four cases, open surgery was performed. Two patients underwent laparoscopy, and one of them had robot-assisted laparoscopy. Partial and complete nephrectomy was done in four and two cases, respectively. Leaks and urinoma were observed in one of the partial nephrectomy cases. As per surgeons’ experience, extensive adhesions and altered anatomy due to hematoma in acute cases made the surgery difficult (Table 5). As compared to the surgically treated group, the angioembolization group had less blood pressure, more pulse rate, less hemoglobin at presentation, and more transfusion requirement. Angioembolization group had larger-sized tumors (Table 6).

In a recent analysis of 28 cases of Wunderlich syndrome, it has been concluded that renal masses are the leading cause of WS, and CT is the diagnostic procedure of choice. Old age is a possible risk factor. Surgical treatment is preferred in patients diagnosed with renal malignancy and in cases of hemodynamic instability (23). All these findings are comparable with our study.

There are a few limitations of our study. First, it was a retrospective single-centered study. The sample size was small. Second, we did not get any biopsy in those cases managed with selective angioembolization. Third, only imaging criteria were used to diagnose those patients with AML. Fourth, we did not encounter any cases of fat-poor AMLs. So, the diagnostic efficacy of CT scan in those cases is debatable. Fifth, no specific criteria were used for the patients to undergo angioembolization or surgical management.

Despite these limitations, this study can be considered significant as WS is not a common disease. WS due to AML is even less common considering that AML constitutes only <1% of the renal tumors. The preexisting literature is mostly case reports and small case series. Therefore, this study can be used as a reference in managing those patients with renal AML presenting with WS in the future. However, further studies with large sample sizes will be useful.

## Conclusion

Wunderlich syndrome is a rare and life-threatening complication in patients with large angiomyolipoma. CT scan plays an important role in its diagnosis. Judicious optimization, angioembolization, and prompt surgical intervention will help deliver better outcomes. NSS can be attempted in cases where renal preservation is of paramount importance.
